# Mental health service user experiences of targeted violence and hostility and help-seeking in the UK: a scoping review

**DOI:** 10.1017/gmh.2017.22

**Published:** 2017-12-11

**Authors:** Sarah Carr, Jessica Holley, Trish Hafford-Letchfield, Alison Faulkner, Dorothy Gould, Christine Khisa, Claudia Megele

**Affiliations:** Department of Mental Health, Social Work and Integrative Medicine, Middlesex University London, Hendon Campus, The Burroughs, London NW4 4BT, UK

**Keywords:** Disability hate crime, interpersonal violence, mental health service user experience, mental health policy and systems, targeted violence and abuse, UK

## Abstract

**Background.:**

The aim of this research scoping review was to assemble an evidence base for the UK on mental health service user experiences and perspectives on mental health-related targeted violence and hostility (‘disability hate crime’). It also aims to address some of the gaps in the knowledge on risk management, help-seeking and prevention from the perspectives of those who experienced targeted violence and hostility because of their mental health problems or psychiatric status.

**Methods.:**

Seven key mental health and social care bibliographic databases were searched for relevant UK research studies from 1990 until 2016. Grey literature was identified through online searches. A scoping review charting approach and thematic analysis methodology were used to analyse the studies.

**Results.:**

In total 13 studies were finally included, over half of which used survey methods. All studies included people with experiences of mental health problems. The studies provide information on: the types of potential hate crime; indicate where incidents take place; give some insight into the victims’ relationship with the perpetrators; the location of incidents as well as the psychological, social, financial and physical impacts on the victim; the types of help-seeking behaviours adopted by the victims; a range coping strategies that people with mental health problems adopted in response to experiences of targeted violence or abuse.

**Conclusion.:**

This scoping review provides a UK-based overview of mental health service user concepts and experiences of mental health-related targeted violence and hostility (‘disability hate crime’). It reveals some specific issues relating to mental health and disability hate crime. Further investigation into disability hate crime with a specific focus on mental health is required. This is a UK-based overview, which offers a useful comparator for researchers, practitioners and policy-makers internationally.

## Introduction

While always an issue of concern, particularly since deinstitutionalization in the UK (Thornicroft, 2006), violence, hostility and discrimination against people with mental health problems have been increasing in prominence in international research and policy over the last 10 years. One recent study investigating the social effects of the 2008 economic crisis on people with mental health problems in 27 European countries found that ‘times of economic hardship may intensify social exclusion of people with mental health problems’ (Evans-Lacko *et al*. 2013, p. 1). The UN Human Rights Council's ‘Report of the Special Rapporteur on the right of everyone to the enjoyment of the highest attainable standard of physical and mental health’ is clear that ‘persons with psychosocial disabilities continue to be falsely viewed as dangerous, despite clear evidence that they are commonly victims rather than perpetrators of violence’ (UN Human Rights Council, 2017, p. 7). Accordingly, the report recommends that States ‘take policy and legislative measures on the prevention of violence in all environments where people live, study and work’ (UN Human Rights Council, 2017, p. 20). Therefore, violence, hostility and discrimination against people with mental health problems have been prioritised as a human rights issue of global concern.

This paper presents a body evidence on the topic from the UK deriving from the scoping review stage of a larger service user researcher-led (Beresford & Croft, 2012) qualitative study set in England entitled: ‘Keeping control: Exploring mental health service user perspectives on targeted violence and hostility in the context of adult safeguarding.’ The study has been designed in the context of State legal and policy reforms in the UK concerning adult safeguarding (DH, 2014). These reforms determine that adult safeguarding should be less reactive and mechanistic, and more about achieving the best outcomes for the individual concerned and responsive to the person and their specific circumstances. Policy implementation work found that ‘using an asset-based approach to identify a person's strengths and networks can help them and their family to make difficult decisions and manage complex situations’ (LGA, 2013). In the UK ‘adult safeguarding’ is defined as: ‘working with adults with care and support needs to keep them safe from abuse or neglect. It is an important part of what many public services in the do, and a key responsibility of local authorities. Safeguarding is aimed at people with care and support needs who may be in vulnerable circumstances and at risk of abuse or neglect. In these cases, local services must work together to spot those at risk and take steps to protect them’ (DH, 2014).

Sin *et al*. (2011) use the term ‘targeted violence and hostility’ against disabled people, which in UK legal terms is categorised as ‘disability hate crime’. In the UK ‘hate crime’ is defined as ‘any criminal offence, which is perceived, by the victim or any other person, to be motivated by a hostility or prejudice based on a personal characteristic.’ (HM Government, 2013). For the purposes of the larger study, the ‘personal characteristic’ is having a mental health problem (also defined as a disability in the UK Equality Act 2010). It is well documented that disabled people, particularly people with mental health problems, are at higher risk of being victims of targeted violence and hostility, although effective evidence-based prevention and protection strategies remain lacking (Emerson & Roulstone, 2014; Mikton *et al*. 2014). This paper will largely use the terms ‘hate crime’ and ‘targeted violence and abuse’ to describe the incidents reported in the studies, with the acknowledgement that victims may not describe or recognise their experience as a disability/mental health related ‘hate crime’, and professionals may not classify or recognise it as such. The discourses on adult safeguarding and risk, mental health and ‘disability hate crime’ have appeared to remain largely separate in research, policy and practice, and overall, mental health service user experiences remain under-researched. The larger study, of which is scoping review is an element, aims to address this situation.

## Scoping review questions and objectives

The scoping review addresses the core components of the main study inquiry and builds on the literature review on risk and safeguarding in UK adult social care Mitchell *et al*. (2012). Among other things, they found that there were a significant gap in the UK primary research evidence on mental health service users’ views and experiences of risk and safeguarding.

The main aim is to ‘map rapidly the key concepts underpinning a research area and the main sources and types of evidence available’ for the UK (Arksey & O'Malley, 2005, p. 194).

The scoping review research questions focus on what is known from the existing UK literature about the following:

- mental health service user concepts and experiences of mental health-related targeted violence and hostility (‘disability hate crime’), risk, prevention and protection
- where mental health service users go to get support if they are frightened, or have been victims of, targeted violence and hostility because of their mental health problem or psychiatric status (help-seeking behaviour)
- responses of adult safeguarding agencies, mental health services and other organisations to mental health-related targeted violence and hostility (‘disability hate crime’) against people with mental health problems because of their mental health problem or psychiatric status
- the key concepts underpinning the literature in the research area and the main sources and types of evidence available
- what and where are the main gaps in the literature

The objectives of the scoping review were to:

- systematically search for peer-reviewed journal papers and ‘grey literature’ that addresses the key research questions;
- assess the types and quality of the included literature (as a determinant of the strength of the reported evidence);
- conduct an analysis and thematic synthesis of the literature identified to address the key research questions and to inform the larger research study investigation;
- assemble an evidence base on adult safeguarding that focuses on mental health service user experiences and perspectives on mental health-related targeted violence and hostility, risk management, help-seeking, prevention and protection.

## Methods

The six-stage methodological framework for scoping reviews, as developed by Arksey & O'Malley (2005) and further refined by Levac *et al*. (2010), was used to conduct and structure the literature review. The six stages are as follows:

1. Identifying the research question
2. Identifying relevant studies
3. Study selection
4. Charting the data
5. Collating, summarising and reporting the results
6. Consultation with expert/stakeholder advisory group

Peer-reviewed English language journal papers were identified via the relevant health and social care databases available at Middlesex University through a keyword search using terms relating to mental health, adult safeguarding, service users, disability hate crime and targeted violence and hostility. In total, seven databases were searched for the years 1990–2016: CINAHL; PsycINFO; Medline; Social Care Online; Emerald; BNI; Cochrane reviews.

Searches for English language ‘grey literature’ was also conducted via online searches (Summon, Google Scholar, Open Grey, relevant UK government websites and organisational websites such as Mind, Mental Health Foundation, Shaping Our Lives, National Survivor User Network, Joseph Rowntree Foundation, Victim Support, SPRU, NIHR SSCR, Disability Archive UK, SCIE, EHRC, BASW), contact with topic experts, key organisations and via team and advisory group member networks.

Hand searching of key journals (i.e. The Journal of Adult Protection) and examinations of article reference lists (particularly previous literature reviews) was undertaken.

The search strategy included both primary and secondary search terms (see Box 1: Primary research terms).

Box 1. Primary search terms

In order to search for mental health service users’ experiences of hate-crime, targeted violence and hostility the following search terms were used:

mental* OR mad* OR psychiatr* OR disab
AND
service user OR survivor OR consumer OR client OR expert by experience OR lived exper* OR patient
AND
views OR experience OR perspectives OR narratives
AND
violence OR abuse OR hate crime OR hostil* OR danger* OR risk* OR victim* OR crime OR bully* OR harass*
Secondary search terms

In the context of the searches retrieved through the primary search terms mental health service users’ help-seeking behaviours and their experiences of support and safeguarding were searched for using the following search terms:

adult safeguard OR vulnerable adult* OR protect* OR safe* OR prevent* OR peer support
OR
resilience* OR coping OR help* OR managing OR protect* OR support

Inclusion and exclusion criteria were used to eliminate studies that did not answer the research question and to ensure a consistent approach between scoping team members (see Box 2: Inclusion and exclusion criteria). Rather than adhering to a hierarchy of evidence approach, based on methodology, we included empirical studies (quantitative and qualitative) most likely to answer our research question (Aveyard, 2007). However, the methodological quality of included studies was assessed by their using appropriate critical appraisal (CASP) checklists, which are series of questions designed to help reviewers interrogate the quality and reliability of various types of health and social care research, including qualitative studies (CASP, no date).

Box 2. Inclusion and exclusion criteria

*Inclusion criteria*

- Empirical studies conducted in the UK published in peer reviewed journals addressing the areas in the key research questions that have adults (18–65) and/or older people (65+) with mental health problems in their population.
- Systematic reviews and other research reviews published in peer reviewed journals (or peer reviewed formats such as Cochrane or Campbell) addressing the areas in the key research questions that have adults (18–65) and/or older people (65+) with mental health problems in their population.
- Conference papers addressing the areas in the key research questions published in peer reviewed journals or as ‘grey literature’ conference proceedings.
- Mental health service user and survivor research addressing the areas in the key research questions published in peer reviewed journals or as ‘grey literature’.
- Research reports or research reviews from key organisations working in the areas of mental health, hate crime and adult safeguarding, including user-led organisations and initiatives and voluntary and community sector organisations.
- Individual narratives that do not use a recognised qualitative method.
- English language publications.
- Material published between 1990 and 2016.

*Exclusion criteria*

- Duplications.
- Non-UK studies.
- Studies that do not include mental health service user experiences.
- Studies concerning children and young people (0–18).
- Studies concerning dementia or brain injury.
- Commentary pieces.
- Policy and guidance documents.

The study data were synthesised according to the scoping review ‘charting’ approach developed by Arksey & O'Malley (2005). Their ‘data charting form’ the following key information was recorded about each study (see Table 1: Data charting form and numerical in-text reference key), including:

- Author(s), year of publication, study location
- Intervention type, and comparator (if any); duration of the intervention
- Study populations
- Aims of the study
- Methodology
- Key findings or important results

**Table 1.** Data charting form and numerical in-text reference key

Reference	Study aims	Setting and study population	Methodology	Key findings
1. Berzins *et al*. (2003)	To measure levels and experience of harassment experienced by people with mental health problems in the community in Scotland and compare them with the general population.	Community mental health teams. Sampled from four main areas: cities, towns, peripheral housing estates and scattered rural settlements. People with severe and enduring mental health problems between 25 and 65 years old (people with dementia and homeless people were excluded). In the general population those who has been in contact with mental health services within the past 10 years were excluded.	Mixed methods study (collected quantitative and qualitative data in interview). Purposive sample of mental health group on basis of sampling criteria, matched GP group on the basis of criteria. Face to face structured interview to explore experiences of harassment. Allowance of less-structured responses to explore incidence in more depth. Interview schedules developed through focus groups involving service users from voluntary and statutory services. Information gathered on what harassment consisted of, where it took place, who committed it, why it was felt to be occurring and what impact it had upon them. Comparisons between quant data analysed using chi-squared test, qualitative data analysed using Nudist software.	*Participants*: 330 people interviewed (165 people with mental health problems and 165 from general population). Detailed demographics included in paper (age, gender, ethnicity, employment status, living status). *Findings*: 60% of people with mental health problems experienced harassment compared to 44% from general public. (*p* = 0.004). Age had significant influence on whether people with mental health problems experienced harassment (not case for general public). Significant relationship between harassment and both groups who lived in local authority accommodation. *Features of harassment*: verbal abuse most common (those with mental health problems having problems exposed); unwanted interference including false accusation to LA's and telephone calls. Other features included physical threats and actual assaults on occasion. *Who was committing it*: teenagers and neighbours most common across both groups. Teenagers particularly under influence of parents and peers when verbally abusing people with mental health problems. 21% of people with mental health problems experienced harassment from family whereas none from general public reported from family. This harassment was due to being taken advantage of for welfare benefits or medication. Also called names due to mental health problems. *Reasons being harassed*: substantial differences. Majority of people with mental problems thought it was because of mental health problems. General public saw it as fulfilling need of harasser (get money or something to do). *Reporting harassment*: 71% general public reported to police compared to 43% of people with mental health problems. Mental health problems fear will not be taken seriously. Not wanting contact due to negative experiences in past (if under the Mental Health Act 1983). Majority in both groups reported to more than one agency. Mental health group to medical/support staff. Majority in both groups said reporting made no difference. *Strategies to stop harassment*: 46% mental health group took no action; 30% people from mental health group had moved house whilst 16% tried to reason with harasser. *Impact*: almost all mental health group referred to adverse effect it had upon their mental health. Half of GP group also experienced adverse effect on mental health. Mental health group more likely to report fear compared to GP group who were annoyed. *Prevention*: both groups reported education (about mental health problems or of the impact of anti-social behaviour).
2. Chakraborti *et al*. (2014)	To examine people's experiences of hate, prejudice and targeted hostility; to understand the physical and emotional harms suffered by individuals and their families; and to identify ways of improving the quality of support available to victims.	People over the age of 16 who had experienced hate crime.	Mixed methods approach. Online and hard-copy surveys (translate into eight different languages). In-depth, semi-structured face-to-face interviews. Personal and reflective researcher field diary observations.	*Participants*: 1106 questionnaires were completed by people over the age of 16. In total134 people identified as disabled from sample of which 28% (37) were targeted because of mental ill-health. 374 victims were interviewed (no idea of number who had mental ill-health). The profile of research participants was extremely diverse in terms of age, gender identity, ethnicity, refugee and asylum status, religion, sexuality and disability. *Key findings*: 46% of those victimised because of their mental ill-health stated that being a victim of targeted hostility had made them feel suicidal, and 41% had turned to alcohol. 88% of respondents victimised because of their mental ill-health, physical disabilities or learning disabilities were very concerned about becoming a victim of violent crime in the future. 72% of people who reported ill mental health were more likely to feel vulnerable after hate crime committed as opposed to overall sample. Also more likely to feel suicidal after hate crime. Those who were from ethnic minority groups experienced hostility from those who shared same ethnicity or faith as them. This was due to particular identity markers and form of difference. Poor level of support from police was commonly reported because of who they were. Those with a disability (non-mental health specific) were more likely to report crime to other supports i.e. Social workers, nurse or doctor, housing association.
3. Faulkner. (2012)	Giving voice to service users’ fears and concerns about risk Identify additional risks to those commonly identified by professionals and policy-makers Explore differences in perceptions of risks and rights between service users and professionals	Community.	Qualitative research. Sampling: A selection of people through networking programme manager and the author. Individual interviews.	*Participants*: 17 people include disabled people, older people, people with learning difficulties and people with mental health problems. By observation only, the majority may be described as white British. Two people were Black African Caribbean. *Risk of abuse*: current awareness of accusations directed at disabled people being ‘benefits scroungers’ or as ‘faking it’. Heightened risk of taking part in community because of hate crime. People with learning difficulties are at particular risk of bullying and abuse in the community. *Fear of institutional and interpersonal abuse*: mainly within residential settings. Unpleasant treatment, a constant weighing up process as to whether they should speak up about their rights out of fear and who are consequently not receiving the care that is right (e.g. in residential care keeps money that they are entitled to, not getting choice on what you buy, fear of asserting your rights and consequences if you do).
4. Kai & Crosland. (2001)51	To explore experiences and perceptions of healthcare of people with enduring mental ill health.	Four general practices (referring to two consultant psychiatric-led community mental health teams linked to local hospital inpatient unit). Patients with enduring mental health problems. *Other inclusion criteria*: including inability to fulfil roles such as holding down a job; participating in recreational activities; impairment of social behaviour (hallucinations/delusions; violence towards others and self); a mental health diagnosis; *excluded* if had dementia or other organic brain disorder, LD or under 16.	Qualitative design Theoretical sampling In-depth interviews- discuss and reflect upon experiences of healthcare. Broad topics were used as prompts. Grounded methodology. Themes were identified by open coding of key categories.	*Participants*: 32 patients. All receiving continuing care from primary care teams. Selected key themes relevant to scoping review: *Experience of social exclusion*: taking control of their lives and mental health was jeopardised by experiences of victimisation and crime. Most had experienced victimisation where they lived, attributed to their apparent differences to others. Led to feeling fearful about people finding out about mental health problems. Experience both verbal and physical abuse within their local community. Fear of people finding out led to social isolation. Social isolation exacerbated by fear of crime, reluctance to go out as had experienced of crime and burglary. *Contribution of professional care*: valued positive therapeutic relationship with professionals within the context of social exclusion and their need to protect their anonymity about their ill mental health (as able to discuss issues/problems).
5. Kelly (1999)	A part of Kelly's thesis (1999) which explored quality of life of peoples with enduring forms of mental illness.	In the community. People who met the criteria for severe and enduring mental illness (the House of Commons Report, 1994).	Mixed methods. Random sampling strategy. Structured questionnaire based on Quality of Life profile (developed and tested for the study) with follow up comments made by participants. Participants interviewed in their own homes.	*Participants*: 160 respondents. Findings of this discussion paper based upon participants’ responses in relation to one of the components of the QoL structured questionnaire asking people to identify from a list of problems what they have experienced whilst living in the community in the past year namely: broken windows; damp/condensation; mice/rats; poor heating; and harassment. 60% of respondents reported harassment (*n* = 100) Experiences ranged from minor (e.g. children knocking on door and running away to more serious being pushed, jostled or threatened.) Acts of harassment categorised into 3 broad themes: *Harassment while at home*: children and teens banging on their door. Fourteen people reported being subjected to taunts and name-calling while in their own homes (i.e. being sung at from outside their window). Six female respondents reported pornographic material being pushed through their door.One participant reported lit matches being put through their door. Eight reported windows being broken; 15 stones being thrown at windows and doors; 5 reported graffiti (ie. pervert; paedophile). Three found urine or faeces outside their flat; 1 reported bin being emptied outside his flat (followed by complaints by neighbours to council); *Perpetrators of home incidents*: majority reported as the local children. Some adults and teens. *Reasons for harassment*: in some instances harassment was carried out to force person out of their house. Children model stigmatising attitudes and behaviours of adults. Giving up tenancy and leaving home due to neighbours’ harassment. *Harassment on the street*: 12 reported regular harassment for no reason outside of their homes. Often in the form of name-calling; taunting; and sometimes verbal abuse. Seven reported having stones thrown at them or being jostled whilst out running daily errands. *Perpetrators*: children and also significant proportion carried out by teens and adults. *Financial exploitation*: Eleven people reported not doing their own shopping because of harassment or inability. People paid between £5 and £10 for others to do their shopping (but were not aware of being able to place a weekly order by telephone free of charge). Four people reported being accosted after picking up benefit claims. Some reported giving money to persistent beggars due to the location of their accommodation. Seventeen reported neighbours regularly borrowing money or cigarettes and never repaying. Several reported using unofficial home-help services that were above good value. Most blatant financial exploitation was by female neighbours who befriended male and got him to buy register for a mail order catalogue. They ran up a large bill and never repaid him. *Reporting harassment*: some noted how they chose to ignore harassment as ‘it would make it worse’. Reluctance to report harassment. Also did not report it to police as the culprits run away and return when police leave not prepared to report to police or mental health professionals as they would not be believed or they would think they was ill again. 1 man chose not to report to police because the only contact he had with them was when being escorted to hospital (previous negative experiences). Majority are prepared to suffer harassment in silence. *Coping mechanisms*: avoid situations likely to experience harassment. Reclusive lifestyle resulting in only leaving house when absolutely necessarily (e.g. one person had not left house in 8 months). Three people said they carried a weapon with them when going out (i.e. pocket knives, sock with a rock in it).
6. Langan & Lindow (2004)	Provide information on the involvement in risk assessment and management of mental health service users who are considered by professionals to pose a risk to other people.	One urban area in England who were inpatients at two hospitals within the same MHT. *Inclusion*: People who were being discharged from in-patient treatment and moving into the community. Aged between 18–65 years old. Living in set geographical area whom they considered to be a possible risk to others. *Exclusion*: some service users could not be asked as the service users did not know that they were considered risk to others.	In-depth interview with service users at point of discharge (phase 1), and six months later (phase 2). Purposive sampling, psychiatrists who selected people on the basis of the criteria. At each time point, also interviewed three mental health professionals, as well as a relative and a friend. Professional interviewed included psychiatrists, CPNs, social workers, psychologists, housing workers and day care staff.	*Participants*: 129 interviews conducted. 17 service users took part (2 female; 4 from ethnic minority group). Only 14 were interviewed at phase 2. *Key theme relevant to scoping review*: Risk or harm that people experienced from others. Four service users were seen as behaving in ways that increased the risk that they would be attacked by others - for example, preaching to people or being aggressive or confrontational to others when experiencing psychosis. *Differences in perceptions of risk*: service users reported defending themselves as a result of being provoked rather than making unprovoked threats. The differing accounts revolved around whether this service user was being threatening or trying to protect himself (albeit in a threatening way) due to fear. *Insecurity in accommodation*: two had been at the receiving end of harassment by neighbours.
7. MIND (2007)	To explore the extent of fear, crime, and victimisation to which people with mental distress are exposed, and to uncover barriers people face in accessing criminal justice agencies.	Voluntary organisations (Mind associations) People with experience of mental distress.	Mixed methods Short questionnaire (both closed and open ended questions) sent to 2000 people. Assessed attitudes towards personal safety and the role of agencies Two focus groups using vignettes. Explored issues of isolation, exploitation, protection and empowerment and the role of social workers and other agencies to keep people safe from abuse.	*Participants*: 84 completed surveys (response rate of 3.6%); 10 participants took part in a focus group (five in each) and were mixed in terms of age, gender, ethnicity, diagnosis. *Survey key findings*: 84% felt vulnerable or at risk of abuse some or all of the time. Only 16% did not feel at risk. Anecdotal evidence of abuse perpetrated by: family; friends; neighbours; carers; health professionals; care home staff. 86% respondents felt they were responsible for keeping themselves safe; 55% health professionals; 43% family; 37 and friends; 35% police. Disempowering and excluding from decisions about risk; lack of systematic approach to safeguarding which is dealt with internally (rather than referred to police or SG teams); discrimination at heart of criminal justice system results in abuse not being reported by victims (not believed).
8. Pettitt *et al*. (2013)	To understand experiences of victimisation and engagement with the criminal justice system among people with mental health problems.	Community mental health services in London. People with severe mental illness (SMI) based in community mental health teams (CMHTs) for one year or over. 18–65 years old with any diagnosis. Care needed to be planned using Care Programme Approach. Excluded those whose were too ill to consent and whose English language was limited. Those from general population over the age of 16 living in London.	Mixed methods research study. Random sampling strategy for quant research in local London MH Trusts. For qual, invitations were circulated to individuals who had been a victim of crime in the past 3 years. Recruited from local Mind and Victim Support services as well as CMHTs. Quantitative survey- computer based questionnaires modified version of the Crime Survey for England and Wales (CSEW) was used. Compared to that of the general population who took part in the survey over the same period of time in London. Qualitative semi-structured interviews with service users. Data analysed using thematic analysis. Focus groups and interviews also conducted with 30 relevant professional from range of different background i.e. police officers and mental health coordinators.	*Participants*: Quantitative survey: 361 people with SMI responded to the survey. Comparison sample is 3138 people. 60% schizophrenia and 20% bi-polar or depressive disorder. A majority had been ill for more than 10 years and more than half had been admitted under mental health Act1983. Sample was mostly male and Black/Black British ethnicity. Greater personal and area deprivation of 72% *v.* (43% from comparison) were unemployed 63% (*v.* 21%) were council tenants 52% (*v.* 27%) and lived in most deprived areas. *Quantitative survey findings* (comparisons to general population): *Targeted crime*: 43% felt crime was motivated by race, age, sex, disability which was 8 times more likely than control group. A majority of crimes took place in the home, followed by public places. 9% described incident happening in health facility. *Impact of victimisation*: more likely to perceive the crime as serious compared to general population. 98% said they had emotional or mental health problems following the crimes. Also social problems (financial loss/relationship breakdown). More likely to be physically injured but 70% less likely to seek medical help. *Disclosure*: 45% informed the police themselves compared with 35% of control group (not statistically significant). No difference in progress through criminal justice system however, SMI victims less satisfied and less likely to describe police as respectful. 40% did not disclose their experiences to mental health professionals. A third to disclose experiences to their police or mental health professionals. *Help received and wanted*: SMI victims 13 times more likely to receive help than those without SMI. Less likely to receive crime prevention advice compared with 35% of control group. Less likely to make changes following events than control group. Most likely to seek support from mental health professionals and family and friends. Unmet needs were most high in seeking practical of financial help (60%), talking help (40%) and help with accessing CJS (40%). *Participants*: Qualitative interviews: 81 individuals were interviewed. 82% sample lived in London. 57% women and 43% men. 78% were aged between 25 and 54 years. Over half were White British, 22% Black or Black British, 9% Asian, Asian British and 5% White other or White non-British. 17% described themselves as LGB. 23 and described having another disability as well as mental health problem. Only fifth were in unemployed. Half of sample had experienced depression, third anxiety and a third psychosis. Two thirds were accessing support from CMHTs. *Quantitative survey findings Types of crime*: Commonly experienced assault and harassment. Nine people reported being victims of crime whilst in psychiatric settings and in some cases, the offender were staff. Three fifths of crimes were by people they knew and had existing relationships with. *Perceptions of why they were victimised*: Because they would not be believed and would be easily discredited. People saw them as vulnerable because of their mental health problems. Targeted Mental health problem used as a basis for abuse, e.g. mocking verbally and displaying prejudice towards their mental health problem. *Hate crime*: 14 p's described being victims of hate crime. Motivated by hostility or prejudice towards their mental illness. Incidences on wards- imbalance of power between SU and mental health professional made them vulnerable to abuse. Discredited and could block access to help if they complained. *Factors helping them to report crimes*: decisions being taken out of their hands (someone else alerting police): accessibility of the police; Severity of the crime; A desire to prevent reoccurrence or to protect others *Two thirds of sample described negative experiences reporting to the police*: fear incident will be escalated; being blamed; lack of empathy/respect; dropping cases; lack of info and communication; not taken seriously; not being believed; poor responses to disclosure of mental health problem; prejudice attitudes towards mental health *Three quarters of sample described positive experience from police*: positive responses to mental health problems; caring attitude; taking incident seriously; communication; working with other services. *Enabling experiences of court*: pre-court visits, prep; witness service; special measures; judge/magistrate intervening on their behalf *Poor experiences of court*: seeing the perpetrator and their family/supported; cross-examination in court; not being able to make their point; long waiting times; not being given special measures’ lack of info after the trial. *Enablers to seeking help*: presence of support network; current or prior relationships with services; impact of crime as triggers. *Barriers to seeking help*: fearing response; fear or situation becoming worse; barriers associated with knowing the perpetrator; barriers of services; poor responses by individuals in services; impact of crime as barrier (or emotional and mental health); mental health problems as a barrier. *Sources of support*: Informal: family friend, partner, neighbours, work colleagues. Services: CMHTs, inpatient teams; GPs; emergency services; social care services; housing services; solicitors; probations services; local councillors/MPs; voluntary/community sector services. *Problems with services*: inadequate help provided; inappropriate help provided; disempowering or punitive responses; lack of responsiveness of services; complex cases and a lack of effective multi-agency working.
9. Read & Baker (1996)	Investigate discrimination faced by people with mental health problems, and the extent to which it affects their everyday lives.	Community People with mental health problems who are members of local Mind association or similar groups.	Mixed methods study Questionnaires (with closed and open ended questions) sent out to local Mind associations, Mindlink members, and the UKAN network of independent advocacy agencies.	*Participants*: 778 completed questionnaires. Range of diagnoses including: anxiety, depression, OCD, psychosis, PTSD, agoraphobia, panic attacks, eating disorders, and SADs. 51% women and 49% men. Ages ranged from 18–74. Majority identified their ethnic background as UK (675), others ethnicities included: European, Caribbean, Irish, Indian, Asian and others. *Main findings Daily life in public*: 47% reported having experienced harassment or abuse in public because of mental health problems. (29% shouted at in street; 21% threatened; 14% physically attacked; 16% forced to leave premises). *Daily life at home*: 57% were afraid of being attacked in their own homes, with as many actually being harassed (25% in their own homes; 34% outside in immediate neighbourhood) (e.g. of abuse: burgled, lit matches and put in letter box, others had dog faeces, used condoms and abusive letters stuffed through front door). People described being attacked by neighbours, family and friends, landlords, people in authority (police, staff), other patients. 49% had actually been attacked or harassed (21% by neighbours and other tenants; 20% strangers; 7% by landlords). 26% had been forced to move because of harassment. *Daily life at work*: 38% said they had been harassed and teased at work (16% by manager; 25% colleagues; 6% personnel department; 7% other staff). *Parenting*: 24% of children had been teased or bullied because of their psychiatric condition (by other school children, neighbours).
10. Ryan (2000) 2	To explore the risk management strategies employed by users of mental health services	To mental health sites in the North of England. Service users with a diagnosis of schizophrenia, depression or bi-polar disorder were included. Also recruited people who had been an informal inpatient, detained under MENTAL HEALTH ACT 1983, and those who had never been in hospital.	Qualitative research Quota sampling employed. Semi-structured interviews. Explored underclass, medical disempowerment, vulnerability, threat, self-harm, dependency, self-neglect. Grounded theory employed.	*Participants*: 22 participants took part. Mean age of 48.4 years, nine women and 13 men. nine people had diagnosis of schizophrenia, six bi-polar, seven depression. *Results*: viewed risks as the ‘everyday risks’ they faced such as being teased and ridiculed by people they met, neighbours avoiding them. Previous experiences of assaults had impacted upon people's risk management strategies. Avoidance was a key theme that resulted in people then becoming socially isolated. ‘Many of the users who felt in danger from other people, whether they were friends, other users or people they did not know, became socially isolated as their risk management response was often unassertive and aimed at avoiding conflict.’ Participants also reported ‘doing nothing’ in order to avoid experiencing crime. With regard to self-neglect, participants took the line of least resistance and were therefore exploited by others (i.e. from relatives). ‘In relation to other risks users talked about times when they had been homeless, lost contact with family and friends, been assaulted and verbally abused by people in the street, shunned by neighbours and abused physically, financially and sexually.’ Sometimes sought help from other service users or social workers.
11. Hedges *et al*. (2009)	To explore disabled people's experiences of violence and hostility.		Qualitative research Semi-structured interviews with a number of stakeholders from key organisations including those with disabilities.	*Participants*: 30 disabled people were interviewed either with a learning disability or mental health condition. *Key findings Typology of 8 key types of victimisation*: physical, verbal and sexual incidents, targeted anti-social behaviour, damage to property and theft, school bullying, incidents by statutory agency staff, cyber bullying. *Settings and motivation*: Most likely to occur in the street or home-based setting. But also in colleges, work and public transport. Motivations of perpetrator, threat and vulnerability. Also may see people as lesser than them. *Prevention tactics*: People may re-structure their lives to minimise risks (e.g. longer walking route). Most common strategies were acceptance or avoidance. *Reporting and seeking redress*: tended to report to a third party rather than police however, third parties involvement is under studied (i.e. social workers, housing associations, local authorities, civil justice agencies, voluntary bodies, and others can play.) A need for better joined up inter-agency working. *Barriers to reporting*: physical, procedural and attitudinal barriers of reporting to police. Will be in the wrong. Also may be because of victims relationship with perpetrator, may blame themselves for what happened or just believe it's a part of everyday life.
12. Smyth *et al*. (2011) 74(7)	This research aimed to explore the experiences of social inclusion for mental health service users when engaged in everyday community occupations and to identify possible factors that influenced the service user experience.	Mental Health Trust's rehabilitation service in an inner-city area in UK. Service users from rehabilitation service who were engaged in any type of community occupation and who were able to give informed consent.	Qualitative research Convenience sample Individual in-depth interviews in two parts: (1) inclusion web to identify community occupations; (2) used an interview schedule, which covered three main topics: identifying and describing the experience of community occupations, factors that have an impact on engagement and the participant's feelings of inclusion or exclusion. IPA to analyse data	*Participants*: The eight participants consisted of six men and two women with a mean age (range) of 39 years (32–46 years). *Findings*: The three super-ordinate themes were the outside experience, the internal disability and an active lifestyle. *Environmental features of exclusion*: experienced unfriendly, hostile and bullying reactions from other people due to mental health prejudice, racism or homophobia. Negative experiences of social support provided by mental health services which, at times, felt unsupportive and abusive. *Internal disability, internal features of inclusion and exclusion*: *Stigma and safety*: some people ceased to feel safe in their community and restricted them from engaging in community activities due to dealing with unpleasant memories with experiences of hostility and abuse.
13. Wood & Edwards (2005) 10(2)	This study aimed to compare crimes against mentally ill patients living in the community with crimes against students who have a high life-style risk of victimisation.	Community mental health teams and university population. 40 organisations approached to recruit mental health service users	Quantitative research Questionnaire-based research A 55-item victimisation questionnaire was adapted from the British Crime Survey England and Wales (2000) and the National Crime Victimisation Survey (2000). 20 participating charities were sent 225 questionnaires but only 25 were completed (10% response rate) All statistical analyses were conducted using 0.05 alpha level.	*Participants*: The mentally ill patients (N ¼ 40) consisted of 22 females and 18 males with a mean age of 42.28 years (SD ¼ 11 : 27). Of the mentally ill patients, 32.5% suffered from depression, 15% suffered manic depression, 12.5% suffered schizophrenia, and 12.5% had a dual diagnosis. Individual diagnoses included personality, anxiety, and eating disorders. Patients were mainly White (97.5%), the remainder being Black (2.5%). The student participants (*N* ¼ 80) consisted of 46 females and 34 males. Students were asked if they had ever suffered from a mental illness: none said they had. *Key findings*: Half (50%, *N* = 20) of the mentally ill patients and just over a third (38.75%, *N* = 31) of students reported being victimised at least once. *Offender relationship*: students most likely victimised by strangers whereas mentally ill people likely to be victimised by range of people including family, partners, friends and strangers. Mentally ill patients experienced more frequent to victimization. More likely to experience personal victimisation than students. There was an interaction between gender and group, Fð1; 117Þ ¼ 13 : 33, *p*, :001, h ¼ :10, power ¼ 0.95, revealing that mentally ill females experience more frequent victimisation than did mentally ill males or students of either gender. Those with mental illness more likely to hold more negative attitudes towards police. No differences in amount of incident reported to police But mentally ill population more likely to be dissatisfied. Only mentally ill people felt dissatisfied by the police response due to the way they were responded to on a personal level.

A final column was added to the table in order to make additional comments or notes on the included studies (not included in Table 1).

A basic thematic analysis approach (Braun & Clarke, 2006) was used in order to identify, analyse and report patterns (or themes) that ran between the findings of the 13 finally included studies. The key stages in the thematic analysis were as follows:

1. Becoming familiar with the data.
2. Generating initial codes.
3. Searching for themes.
4. Reviewing themes.
5. Defining and naming themes.
6. Producing the report.

Using the data charting table, an initial list of key findings from each of the included studies was created. These key findings were grouped and merged together in order to develop a list of themes and sub-themes that mapped on to the scoping review's key research questions and objectives.

The findings sections of each of the 13 original papers were re-checked in order to ensure that extracts of data reported on the findings had not been missed thus further refining the emergent themes and sub-themes.

In order to avoid potential bias and subjective decision-making, the papers were re-read by another research team member in order to check whether the first reviewers’ interpretations or conclusions drawn from the included studies’ findings were aligned to the data presented in the original studies. The two reviewers were largely in agreement, and where there was some discrepancy in opinion, the differences were discussed and a mutually agreed position reached.

## Findings

### Search results

**Fig. 1.**
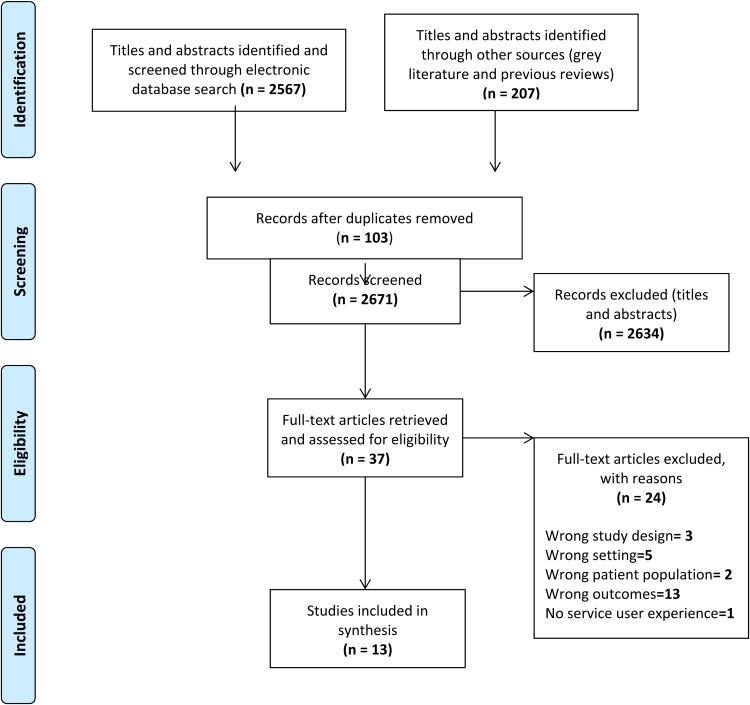
PRISMA flow diagram: The study identification, screening and selection process.

The database, grey literature and previous relevant literature review searches yielded a total of 2774 papers, reducing to 2671 after duplicates were removed. A total of 2634 articles were excluded as they did not meet inclusion. A total of 37 full-text copies were obtained and 13 relevant papers were finally included in the final scoping review (see Fig. 1: PRISMA flow diagram).

The studies are numerically referenced in the text. The numbers and corresponding references are detailed in Table 1: Data charting form and numerical in-text reference key.

Seven out of the 13 studies included in the review explicitly focused on mental health service users’ experiences of hate-crime and targeted harassment, abuse, violence or victimisation (e.g. 1, 7, 2, 11, 13, 8, 5). In the six remaining studies, experiences of targeted violence or abuse were implicit within the wider studies aims and objectives. For example, three studies explored services users’ perceptions, experiences and involvement in risk management (6, 3, 10), two studies explored quality of life and social inclusion whilst living in the community (5, 12), one study explored experiences of discrimination (9) and one study explored experiences and perceptions of healthcare (4).

Over half of the included studies used survey methods (1, 2, 5, 7, 8, 9, 13). With the exception of one study, these studies employed mixed methods approaches by using a variety of qualitative methods alongside the survey data including, open-ended questions (9), focus groups (7), semi-structured interviews (1, 8, 2, 5) and field diaries (2). The remaining six studies employed a qualitative methodology using either semi-structured (10, 11) or in-depth interviews (4, 3, 6, 12). One study also conducted follow up in-depth interviews (6).

Three out of the seven studies that adopted a survey approach used standardised measures (13, 8, 5). Both Wood & Edwards (2005) and Pettit *et al*. (2013) used an adapted version of the Crime Survey for England and Wales. Kelly (1999) used a structured questionnaire based on a quality of life profile that was developed and tested for the study.

With regard to sampling and recruitment, five studies recruited from community mental health teams (1, 12, 13, 8, 10), four from the community (3, 5, 2, 9), two from voluntary services (7, 11), one from general practices (4) and one from inpatient hospitals (6). A purposive sampling strategy was used to recruit participants in four studies (1, 4, 6, 10) whilst other studies used random (5, 8) and opportunistic sampling strategies (12). The sampling strategy was not reported in six of the included studies (3, 7, 2, 11, 13, 9).

All studies included people with experiences of mental health problems. One study also collected data on mental health professionals’ views (8), and in two studies data were collected on the experiences of those with learning disabilities as well as those with mental health problems (11), or encompassed within experiences of all victims from the general public (2). Furthermore, two studies directly compared the experiences of people with mental health problems with the general population (1, 8) and one study compared with students who had high life-style risks (13).

With regard to participant demographics, a majority of studies reported a near even spread of male and female participants (1, 2, 4, 6, 7, 8, 9, 10, 13). In addition, in two studies a small number of the participant sample identified themselves as transgender (2, 11). The age range of participants for most of the included studies were between 18 and 70 years with the average age range in four of the studies being between 40 and 49 years old (13, 1, 7, 12), and between 20 and 29 years old in three of the studies (2, 4, 10). Those studies that reported participant's ethnicity, reported a majority of their sample as White British (2, 3, 6, 5, 7, 8, 9, 11, 13). Some studies did not give specific details on participant demographics, only stated how the participant sample were diverse with regard to, for example, age, gender, ethnicity, religion, sexual orientation and disability (e.g. 2, 3 ,5, 12).

### Review findings

#### Nature of incidents

The types of hate crime experienced by those with mental health problems included verbal abuse (1, 4, 5, 8, 9, 10), physical threats and assaults (1, 4, 5, 8, 9), vandalism of property (5, 9), financial exploitation, (3, 5, 10) and in one study, one participant reported sexual exploitation (10).

#### Location of incidents and relationship to perpetrator

In order to provide more detail on the types of hate crimes, violence or abuse people with mental health problems experience, it is important to describe where the incidents usually took place and the individual's relationship with the perpetrator/s.

Whilst out in their local communities, participants from several studies described how their neighbours, more often teenagers and children, would shout offensive comments and sing abusive chants at them (5, 6, 8, 4). This verbal abuse would usually result in the perpetrators specifically setting out to expose the victim's mental health problems. Some experienced verbal abuse from strangers whilst on public transport (2, 11) whilst in one study, one participant reported physical and verbal abuse by strangers whilst sleeping rough on the streets (10).

Participants in several studies reported experiencing harassment and intimidation from neighbours, landlords and other tenants whilst in their own homes (5, 8, 9, 11). This ranged from children knocking on their door and running away, to various acts of vandalism, such as stones being thrown at windows, graffiti (5) and unwanted content being pushed through their letterbox (i.e. abusive letters, used condoms, pornographic material, lit matches and dog faeces) (9). In Kelly's (1999) study, one participant reported having their bin emptied out onto their front garden only to then be reported to the local authority with false accusations of not keeping their property tidy. This supports findings by Berzins *et al*. (2003) that there was a significant association between harassment and those living in local authority accommodation for both people living with mental health problems and the general public.

According to Woods & Edwards (2005), those with mental health problems are more likely to experience victimisation by people they know as opposed to a student population who are more likely to be victimised by strangers. In some cases, neighbours or other tenants within their supported accommodation would ‘be-friend’ those with mental health problems in order to exploit them, for example, by borrowing money or cigarettes and never paying them back (5, 10). Family abuse was also more likely to be reported by those with mental health problems (Berzins *et al*. 2003). This concurs with several of the studies in which participants described how family and friends would also try to take advantage of their disability allowance (7, 9, 10). In one study, participants described how healthcare professionals would keep hold of their money and not give them a choice about how they spent it (3).

In one survey-based study, 38% of respondents reported having been harassed and teased in the workplace by managers, colleagues and the personnel department because of their mental health problems. These findings were supported by Sin *et al*. (2009) who also reported those with disabilities having experienced victimisation in work and colleges. However, this study did not report details on nature of the victimisation and furthermore, collected data from a non-mental health specific population. Participants in one study reported being victims of crime within psychiatric settings, and in some cases, the offenders were staff (8). Two other studies reported people with mental health problems experiencing victimisation by those in authoritative positions such as health care professionals and police (7, 9); however, there were no details provided on the nature of the offences.

#### Reasons and motivations for attacks or abuse

There were several motives behind perpetrators’ attacks on those with mental health problems. The most common motivation for the violence or abuse to occur was because of noted differences between the victim and the perpetrator. Examples of this included, the perpetrator holding prejudiced views towards those with mental health problems (4, 8), the victim behaving in ways that may increase the risk that they would be attacked by others (e.g. preaching to others; being confrontational when extremely distressed) (6), and the perpetrator seeing them victim as ‘lesser than them’ (11). Some experienced hostility from individuals who were the same ethnicity and/or had the same faith as them, where those with mental health problems were seen as going against specific identity markers held within that specific community (2). In a 2014 study, some participants described being victimised due to recent focuses on the Government's benefits reform whereby they were called ‘benefit scroungers’ by others (2).

In some studies, participants felt that they had been a victim of a crime because the perpetrator knew they could take advantage of the person because of their mental health problems. For example, some victims believed that they were targeted by family and friends who wanted to take their welfare benefits or medication (1, 3, 5). In one study, participants believed they were an easy target as they would not be believed and would be easily discredited due to having a mental health problem (8).

Some victims felt that teenagers and children who committed hate crimes against them were influenced by the inter-generational beliefs of older family members; they, therefore, modelled the stigmatising attitudes of others (1, 5).

#### Impact of targeted violence and abuse

Following experiences of hate crime, violence or abuse participants in several studies reported feeling vulnerable, afraid and unsafe about becoming victimised again in the future (1, 2, 9, 3, 12). In contrast, this study found that the general public sample were more likely to experience anger rather than fear (1). In another study, participants who experienced verbal abuse within their local community were more afraid of others finding out about their mental health problems than the actual incident reoccurring (4).

Participants in one study described how their existing mental health problems had either deteriorated or they had developed new mental health problems after being victimised (1). In addition, nearly half of the participant sample reported feeling suicidal (1).

Some studies indicated other impacts of mental health-related targeted violence and abuse. For example, some people experienced financial loss due to exploitation or loss of their job whereas others’ relationships had broken down (8). In one study, participants reported turning to alcohol abuse (2). Individuals also reported physical injuries following from physical abuse (8).

#### Help-seeking behaviour

People with mental health problems most commonly disclosed their experiences to and sought support from mental health professionals (social workers, nurses and doctors) (2, 7, 10), police (1, 7) or family and friends (7). Other reported sources of support included housing associations, support provider charities (i.e. Victim Support), probation services, solicitors, and local councillors or politicians (2, 8).

According to Pettit *et al*. (2013), there were a number of factors that helped people to report experiences of what could be categorised as a hate crime. These included a desire to prevent reoccurrence or to protect others, having a good support network, having current or prior contact with services, and a positive therapeutic relationship with mental health or other support professionals. For those who did not want to report hate crime incidents, it helped having someone in an advocacy role who could peruse the matter on behalf of the individual (e.g. someone else alerting the police).

In several of the included studies, the police were a common source of potential help for victims. In Pettit's study (2013), participants described several factors that helped them report crimes and seek help from the police, which included them having a caring attitude, taking the incident seriously, communicating effectively and being willing to liaise with other services or professionals (8). In some studies, participants reported dissatisfaction with previous experiences of reporting incidents that could be classified as hate crimes to the police where they were not taken seriously and/or where reporting had made no difference (8, 1, 5). Participants also described how police had lacked sympathy, were disrespectful, or had not supported the person due to negative attitudes towards mental health problems (2, 8). There were cases where participants described how they had not and would not report incidents to the police due to a fear and distrust of the police, which was often described in the context of previous negative experiences with police (i.e. only had contact when having had police involvement with hospitalisation) (8). In another study, some people did not report to the police as they felt that they ‘would be in the wrong’ (11).

There were also various other reasons why participants chose not to report being victimised because of their mental health problems. Some reported feeling afraid that it would make the situation worse (5, 8), or that they would be penalised (e.g. the support they receive would be negatively affected) (3). In one study, participants reported being worried that access to services would be blocked if they made a complaint about abuse on wards by mental health professionals (8). In some cases, participants were unsure of where to go for support and who to seek help from (8). In another study, a majority of participants (86%) felt solely responsible for keeping themselves safe and therefore did not feel the need to report the incident or seek help from others (7).

Whether people reported the incident or crime was, at times, dependent upon the extent to which the victim understood it to be motivated by their mental health status. For example, in one study, some participants did not perceive the incident to be worth reporting because it was either considered to be a part of everyday life or was not seen as mental health-related disability hate-crime due to the nature of their relationship with the perpetrator (i.e. a member of their family) (11).

#### Personal coping strategies

##### Avoidance

There were a number of ways in which people chose to manage and cope with the aftermath of being attacked or abused because of their mental health problem. Participants in several studies most often reported changing their lifestyle in order to avoid similar incidents reoccurring. This ranged from individuals avoiding certain walking routes or places (11), through to avoiding leaving their home altogether (10), with Kelly's study (1999) reporting one participant having not left their home in 8 months. In more extreme cases, people described having to move homes in order to get away from the verbal abuse and harassment they were exposed to within their neighbourhood (1, 5, 9). As a result of these avoidance strategies, participants often described feelings of isolation and social exclusion (3, 4, 12, 5).

##### Self-defence

Some individuals preferred to adopt more proactive coping strategies including attempting to reason with their abuser (1). More specifically, Langan & Lindow (2004) described incidences where victims attempted to retaliate by returning the verbal abuse, but what was seen as self-defence by the victim was often viewed by others as threatening behaviour due to the person's history of mental health problems (6). One individual described carrying around weapons (i.e. pocket knives and a sock with a rock in it) in preparation for the next time that they are attacked (5).

##### Acceptance

Linking to those individuals who chose not to report incidences of targeted violence or abuse, some people chose to accept what had happened to them by doing nothing about it (5, 1, 8). Findings from two studies noted that crime victims with mental health problems were less likely to make changes following the incident compared with victims from the general population (1, 8). Furthermore, participants in one study reported ‘doing nothing’ in order to avoid experiencing similar incidences in future (10).

#### What types of help and support do people need or want?

Reflecting the findings from Mitchell *et al*. (2012), only three of the 13 studies included the perspectives of people with mental health problems who had been victims of targeted violence, hostility or hate-crime on what type help and support is needed. This constitutes a gap in the UK literature. In one study, participants were asked what sort of support would they want or need following from their experiences of hate-crime (8). Participants’ suggestions included crime prevention advice, advice on how to access the criminal justice system and psychological support services such as talking therapies. The need for the delivery of psycho-educational classes for the general public to help stop anti-social behaviour and targeted abuse within local communities was highlighted (1). One study showed that people with mental health problems were not routinely involved in discussions about risk management and safeguarding, which was indicated as a problem (6).

## Discussion

Much of the current general research on adult safeguarding in the UK explores systemic issues, service configuration and models, decision-making and practitioner concepts of safeguarding (Johnson, 2011; Graham *et al*. 2014; Norrie *et al*. 2014; Trainor, 2015). Research suggests that reactive and technical approaches to risk management and safeguarding are inadequate for addressing the complex circumstances and individual needs (Manthorpe *et al*. 2008). There is evidence that organisational and professional concerns about risk and safeguarding could impede progressing UK adult safeguarding practice reforms for people with mental health problems (Carr, 2011). The risk-averse culture within UK mental health services has been found to be disempowering for service users who are unable to be meaningfully involved in the processes of risk management, assessment and decision-making that affect them (Whitelock, 2009; Faulkner, 2012; Wallcraft, 2012).

Literature reviews highlight the absence of all service user perspectives, and particularly mental health service user perspectives, in studies on risk and safeguarding and that risk and safety, are defined by practitioners and articulated using managerial language (Mitchell & Glendinning, 2007; Carr, 2011; Mitchell *et al*. 2012; Wallcraft, 2012). Only one piece of UK user-led research work started to address the issue of absent user voices and revealed that fear was a significant concern for service users, particularly those with mental health problems, but this is not necessarily something considered by practitioners (Faulkner, 2012) or community safety. Discourses from the UK on adult safeguarding and risk, mental health and ‘disability hate crime’ have remained largely separate across research, policy and practice.

Despite limitations on the number of studies, and the types and quality of evidence included in this scoping review, the findings reveal a degree of thematic consistency and important insights into mental health service user experiences of targeted violence and hostility, victimisation or ‘disability hate crime’ and adult safeguarding that begins to address some of the gaps in the knowledge.

The studies provide information on the types of potential hate crime experienced by people with mental health problems, indicate where incidents take place, give some insight into the victims’ relationship with the perpetrators. The small body of research studies examined here gives an overview of the location of incidents as well as the psychological, social, financial and physical impacts on the victim. The included studies also highlighted the types of help-seeking behaviours adopted by the victims; where they seek support and whom they disclose their experience to; the factors that may help or hinder the victim from reporting the crime and/or seeking support through to the types of support that people may want or need. The studies revealed range coping strategies that people with mental health problems adopted in response to experiences of targeted violence or abuse.

From the studies, it appears that people who are targeted for violence and abuse or exploitation because of their mental health status or problems are more likely to have a fear than an angry response, with a tendency to avoid situations perceived to be risky or to self-isolate, and a heightened sense of vulnerability. Some of the reported fear is related to fear of exposure of having mental health problems. This raises a wider concern about factors that prevent the social and community inclusion of people with mental health problems in the UK, and circumstances that increase people's vulnerability. However, the studies raised the question: are people being targeted because someone has an active dislike for them due to their mental health problems OR are they victimised because the perpetrator/s know that someone is vulnerable because of their mental health problems and associated circumstances?

Although none of the studies allowed for a comparator in terms of types of disability, it is notable that people with mental health problems tended to feel that they would not be believed by authorities; that somehow the abuse, violence or exploitation is their fault or is to be accepted as part of daily life; and that some saw themselves as an ‘easy target’ because of their mental health problems. These patterns of ‘psychiatric disqualification’ (where people feel they will be delegitimised or discredited because of their mental health problems: Lindow, 2001), chronically low self-esteem or self-worth can act a serious barriers to victims of mental-health related disability hate crime from recognising abuse, accessing appropriate mental health, police and criminal justice support and from pursuing their right to justice as citizens. Finally, the literature suggests that experiences of violence and abuse relating to mental health can increase the risk of further mental health problems or mental health crisis.

The review revealed some tentative but important findings about the relationship of services and professionals to the issues under investigation. Mental health professionals (including social workers), police or family and friends were most commonly identified as sources of help or the first course for reporting the incident. Adult safeguarding did not feature strongly in the findings about help-seeking behaviour and reporting. Of particular concern was the emerging finding on experiences of mental health-related violence, abuse or victimisation within mental health services and inpatient wards, sometimes by staff. In such cases, victims described being too afraid to report the incident because they believed their mental health support would be compromised or that they would be told they were ‘in the wrong’.

Finally, the included studies outlined the importance of positive therapeutic relationships with mental health professionals; advocacy and liaison or joint working between all involved services; clear communication and caring attitudes from police and mental health professionals; and having the incident taken seriously by the person it is reported to. These offer helpful recommendations for improving mental health and policing practice for victims of mental health-related hate crime, or targeted violence and abuse for the UK and potentially, other countries with similar health and social service policies and infrastructure.

## Study limitations

Although findings can potentially inform developments internationally, this study is limited to the UK. A limitation of the included studies is in relation to the adopted recruitment and sampling strategies whereby three studies either used opportunistic sampling whilst a further six studies did not report any details on the sampling strategy and/or no specific detail on participants’ demographics. The four studies that used a strategic or purposive sampling strategy provided limited detail on the criteria used to inform their sampling framework.

It is therefore difficult to know how representative and varied the findings are to the types of targeted violence and abuse experienced by mental health service users and whether the findings capture the varied sources of support that are sought and coping strategies that are adopted. The lack of data detailing ethnicity, sexual orientation, physical disability, gender and gender identity makes it problematic to assess if and how these characteristics intersect in cases of mental health-related violence and hostility.

A number of the studies included in the review explored the experiences of mental health service users alongside those of the general public. One study examined hate crime and victimisation without breaking down the data to reveal specific findings for people with mental health problems, so it was very difficult to discern the extent to which study findings were specifically relevant to the scoping review question and topic.

## Conclusion

This scoping review provides a UK-based overview of mental health service user concepts and experiences of mental health-related targeted violence and hostility (‘disability hate crime’), risk, prevention and protection; where victims go for help and approach protection and prevention, including what might prevent them from seeking help; and their experiences of the responses mental health services, the police and other organisations to their reporting incidents of violence or abuse relating to their mental health status.

It reveals some specific issues regarding mental health and disability hate crime, particularly relating to victim fear responses, social isolation, ‘psychiatric disqualification’, acceptance of violence or abuse as part of everyday life, stigma and its relationship to help-seeking and the expectation of ‘not being believed’ or ‘being in the wrong’. This suggests that further investigation into disability hate crime with a specific focus on mental health is required; and one that considers other intersecting forms of targeted violence and abuse (such as sexism, racism or homophobia). It also reveals that incidents can occur within mental health settings, with staff cited as perpetrators.

This is a UK-based overview that offers a useful comparator for researchers, policy makers and practitioners in other countries, particularly nations with social policies on safeguarding vulnerable adults. It is well documented that the stigma, discrimination, and abuse experienced by those with mental health problem are a concern for global mental health, with the action being taken on an international level (Thornicroft *et al*. 2008; UN Human Rights Council, 2017). It is likely that there will be commonalities and variations in people's experiences internationally, and comparative national reviews of research are useful for understanding the international picture on the targeted violence and abuse of people with mental health problems and service responses to it.
